# Data for molecular recognition between polyamide thin film composite on the polymeric subtract by molecular dynamic

**DOI:** 10.1016/j.dib.2019.103910

**Published:** 2019-05-03

**Authors:** Wan Zulaisa Amira Wan Jusoh, Sunarti Abdul Rahman, Abdul Latif Ahmad, Nadzirah Mohd Mokhtar

**Affiliations:** a Faculty of Chemical and Natural Resources Engineering, Universiti Malaysia Pahang, Lebuhraya Tun Razak, 26300, Gambang, Kuantan, Pahang, Malaysia; b School of Chemical Engineering, Engineering Campus, Universiti Sains Malaysia, 14300, Nibong Tebal, Pulau Pinang, Malaysia; c Faculty of Engineering Technology, Block A3, Universiti Malaysia Pahang, Lebuhraya Tun Razak, 26300, Gambang, Kuantan, Pahang, Malaysia

## Abstract

This paper focus to examine the best molecular interaction between Polyamide Thin Film Composite (PA TFC) layers with different properties of the support membrane. The support membrane of Nylon 66 (N66) and Polyvinylidene fluoride (PVDF) was chosen to represent the hydrophilic and hydrophobic model respectively in the Molecular Dynamic (MD) simulation. The Condensed-Phase Optimized Molecular Potential for Atomistic Simulation Studies (COMPASS) force field was used with the total simulation runs were set 1000 picoseconds run production ensembles. The temperature and pressure set for both ensembles were 298 K and 1 atm respectively. The validity of our model densities data was check and calculated where the deviation must be less than 6%. The comparison between hydrophobic and hydrophilic of the support membrane data was examined by the distance and magnitude of intensity of the Radial Distribution Function (RDF's) trends.

**Table:** Specifications Table

Subject area	*Physics, Chemistry*
More specific subject area	*Polymer, Membrane*
Type of data	*Table, image, figure*
How data was acquired	*Molecular Dynamic simulation Material Studio (version 7.0) software from Accelrys, Inc.*
Data format	*analysed*
Experimental factors	*The PVDF and N66 were chosen to their good hydrophobicity and hydrophilicity respectively as subtract for TFC deposition. The molecules structures of the polymers were firstly sketched then went through geometry optimization, construct simulation box followed by minimization modules.*
Experimental features	*For each polymer, the repeating unit was first built and its geometry optimized by energy minimization using the COMPASS force field. Then, the configurations were employed an energy minimization process using the followed by NVE (number molecules, volumes, and total energy) and NPT (number molecules, pressure, and temperature) ensemble at* 1 atm*. The trajectories of interaction between molecules were analysed using the Radial Distribution Function (RDF) plot*
Data source location	*Universiti Malaysia Pahang, Kuantan, Malaysia. (Coordinate: 3.718491,103.120784)*
Data accessibility	*All data available within the paper*
Related research article	*T. Araki, R. Cruz-Silva, S. Tejima, K. Takeuchi, T. Hayashi, S. Inukai, T. Noguchi, A. Tanioka, T. Kawaguchi, M. Terrones, M. Endo, Molecular Dynamics Study of Carbon Nanotubes/Polyamide Reverse Osmosis Membranes: Polymerization, Structure, and Hydration, ACS Appl. Mater. Interfaces. 7 (2015) 24566–24575.*

**Table:** 

Value of the data • *MD is the right tool to recognize the compatible monomers selection and to explicate the behaviour of interfacial diffusion and bonding between the TFC layers with the support membranes prior to experimenting work.* • *The data will be helpful to examine the interaction between two molecules to avoid the loose formation of PA TFC layer on the subtract polymers which never proven clearly but only assumptions by the common analytical instrument such as Fourier-transform infrared spectroscopy (FTIR), X-ray powder diffraction (XRD) and energy-dispersive X-ray spectroscopy (EDX) during the operation.* • Evaluation on the TFC membrane formation on the particular subtract by MD simulation provide the interaction data with subtract worth for energy, time and cost saving which provide a better understanding in advance before laboratory work.

## 1 Data

**Fig. 1:**
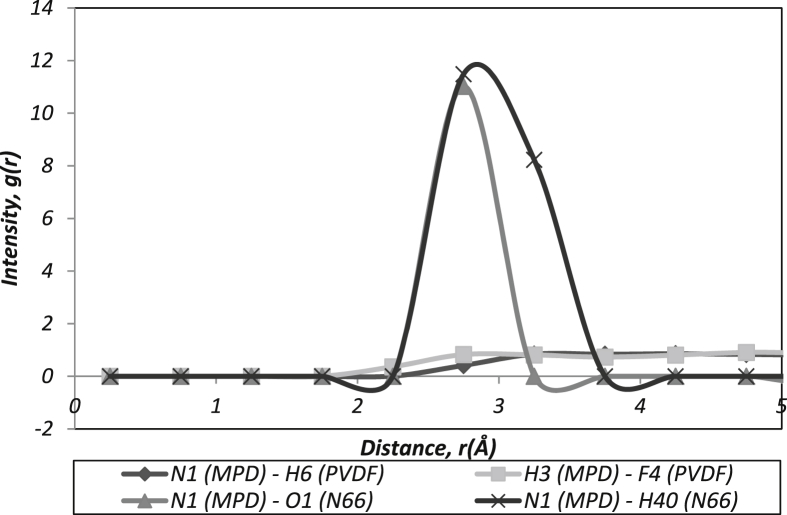
Intermolecular interaction between polymer chains with MPD.

**Fig. 2:**
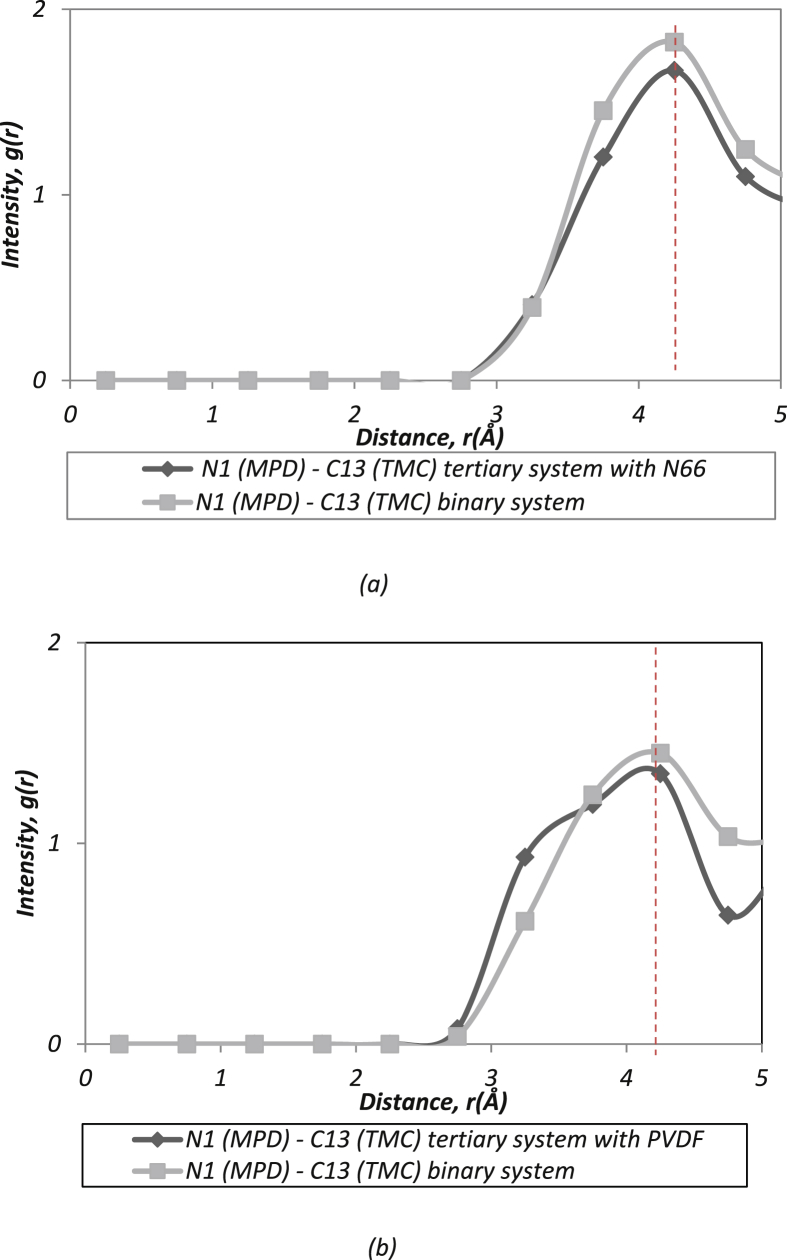
Cross-linking between acyl and amine functional group.

**Fig. 3:**
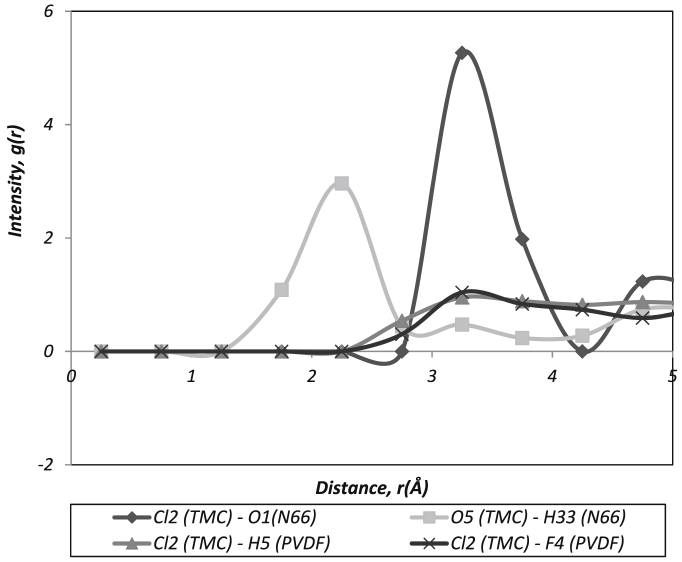
Intermolecular interaction between polymer chains with TMC.

**Fig. 4:**
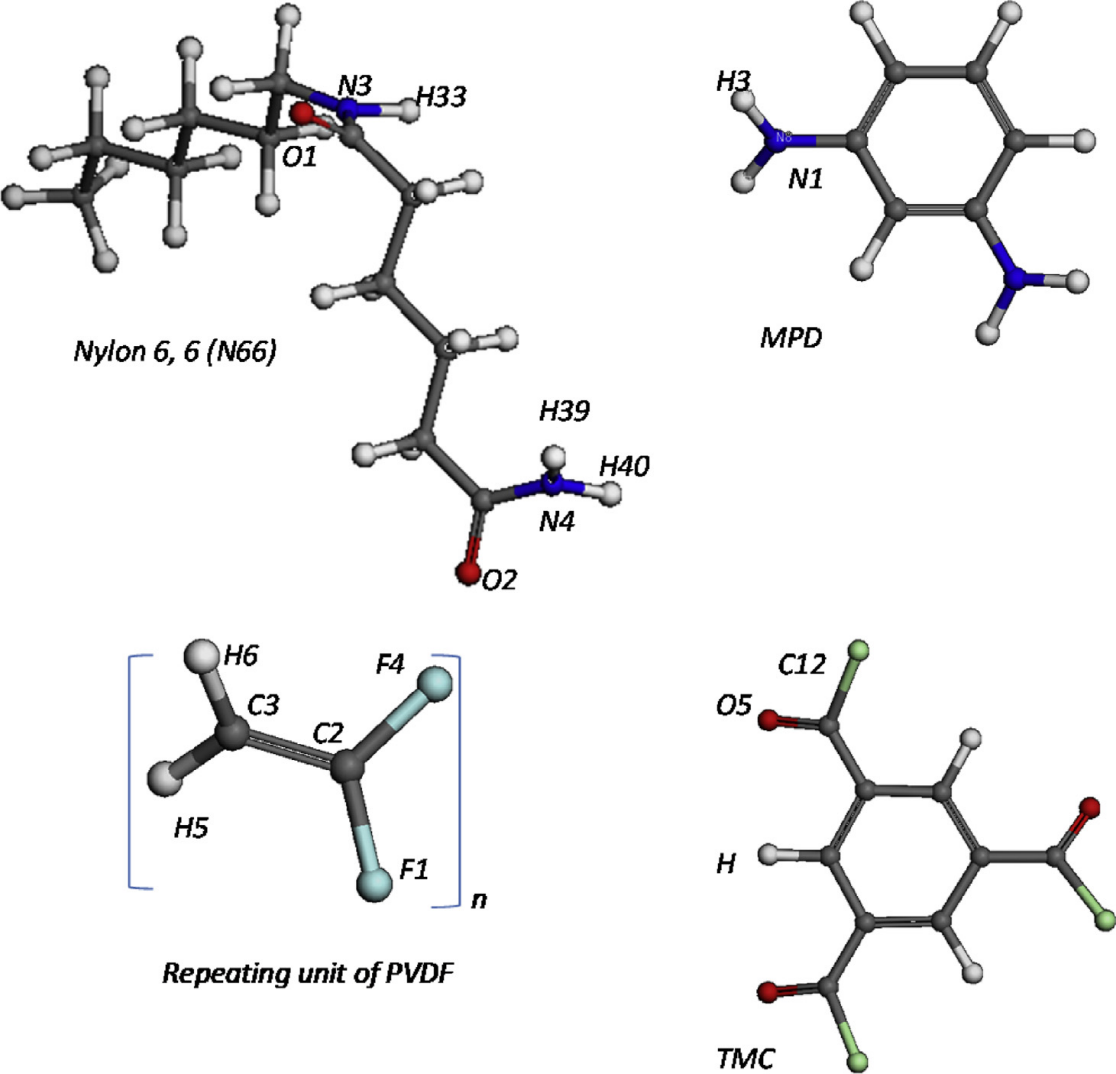
The repeat unit of monomers chain molecule.

MD simulations give insight into the process at the molecular level and to analyse the intermolecular interaction between various monomers concentration on the subtract membrane [1], [2]. In laboratory work, PA TFC is produced by exposing subtracts introduce the amine solution, *m*-phenylenediamine (MPD) first before introducing into organic monomers trimesoyl chloride (TMC) [3], [4]. Thus, in the simulation, the monomers were introduced separately rather than as TFC membrane to mimic real experimental method. In this case, N66 and PVDF were simulated along with MPD and TMC in a tertiary system as shown in Table 1 [5], [6]. In order to make sure the simulation parameters are acceptable, the density initial data setting must be less than 6% error than the final density obtained in the simulations as presented in Table 2. The density of the initial setting was obtained from the previous study [7]. Meanwhile, RDF plot shows the relationship between r which is the distance between atom pairs in each of the trajectory distance of atom with other neighbouring atom and g(r) is the tendency of the atom to interaction/probability to have interaction between atoms [8] as displayed in Fig. 1, Fig. 2, Fig. 3, Fig. 4. The interaction data indicate strong interaction must be in radii of ∼5.0 Å [9]. Fig. 1 displays the main interaction between MPD toward subtracts. Where:

**Table 1:** Simulations setting with the constant temperature.

System	Number of molecules	Equilibrated cell size:A x B x C [Å]
*MPD/TMC*	*10/10*	*18.37 × 18.37 × 18.37*
*N66/MPD/TMC*	*50/10/10*	*23.59 × 23.59 × 23.59*
*PVDF/MPD/TMC*	*50/10/10*	*22.58 × 22.58 × 22.58*

**Table 2:** The deviation of the average simulated densities and temperature.

System	Average Density (g/cm^3^)		Deviation (%)^c^	Temperature (K)		Deviation (%)^c^
	Simulated values a	Setting values b		Simulated values a	Setting values b	
*MPD/TMC*	*1.054*	*1.173*	*5.40*	*299.100*	*298.00*	*0.369*
*N66/MPD/TMC*	*1.050*	*1.050*	*5.00*	*297.928*	*298.00*	*0.024*
*PVDF/MPD/TMC*	*1.160*	*1.175*	*1.28*	*298.025*	*298.00*	*0.008*

a *Experimental value in Ref.*[13].

b Predicted by simulation.

c Deviation = [(simulated value – setting value)/setting value ]x 100.

N (MPD) – O (N66): Intermolecular interaction between nitrogen in the MPD molecules with oxygen atom in the N66 group.

N (MPD) – H (N66): Intermolecular interaction between nitrogen in the MPD molecules with hydrogen in the N66 group.

H (MPD) – F (PVDF): Intermolecular interaction between hydrogen in the MPD molecules with fluoride in the PVDF group.

N (MPD) – H (PVDF): Intermolecular interaction between nitrogen in the MPD molecules with hydrogen in the PVDF group.

*Meanwhile, interfacial polymerization reactions between MPD and TMC produce the medium RDFs data trends in* Fig. 2*. The interactions of binary system (MPD/TMC) were comparing with both the tertiary system, (N66/MPD/TMC) and (PVDF/MPD/TMC)* [10]*. Where:*

N (MPD) – C (TMC): Intermolecular interaction (crosslink) between nitrogen in the MPD molecules with carbon in the TMC group [11], [12].

*There were also interactions detected between TMC-subtracts presented in* Fig. 3 *which contributes to the better attachment of TFC layer ont*o support membra*ne. Where:*

Cl (TMC) – O (N66): Intermolecular interaction between chlorides in the MPD molecules with the oxygen atom in the N66 group.

O (TMC) – H (N66): Intermolecular interaction between oxygen in the MPD molecules with the hydrogen atom in the N66 group.

Cl (TMC) – F (PVDF): Intermolecular interaction between chlorides in the MPD molecules with the fluorine atom in the PVDF group.

Cl (TMC) – H (PVDF): Intermolecular interaction between chlorides in the MPD molecules with the hydrogen atom in the PVDF group.

## 2 Experimental design, materials and methods

**Fig. 5:**
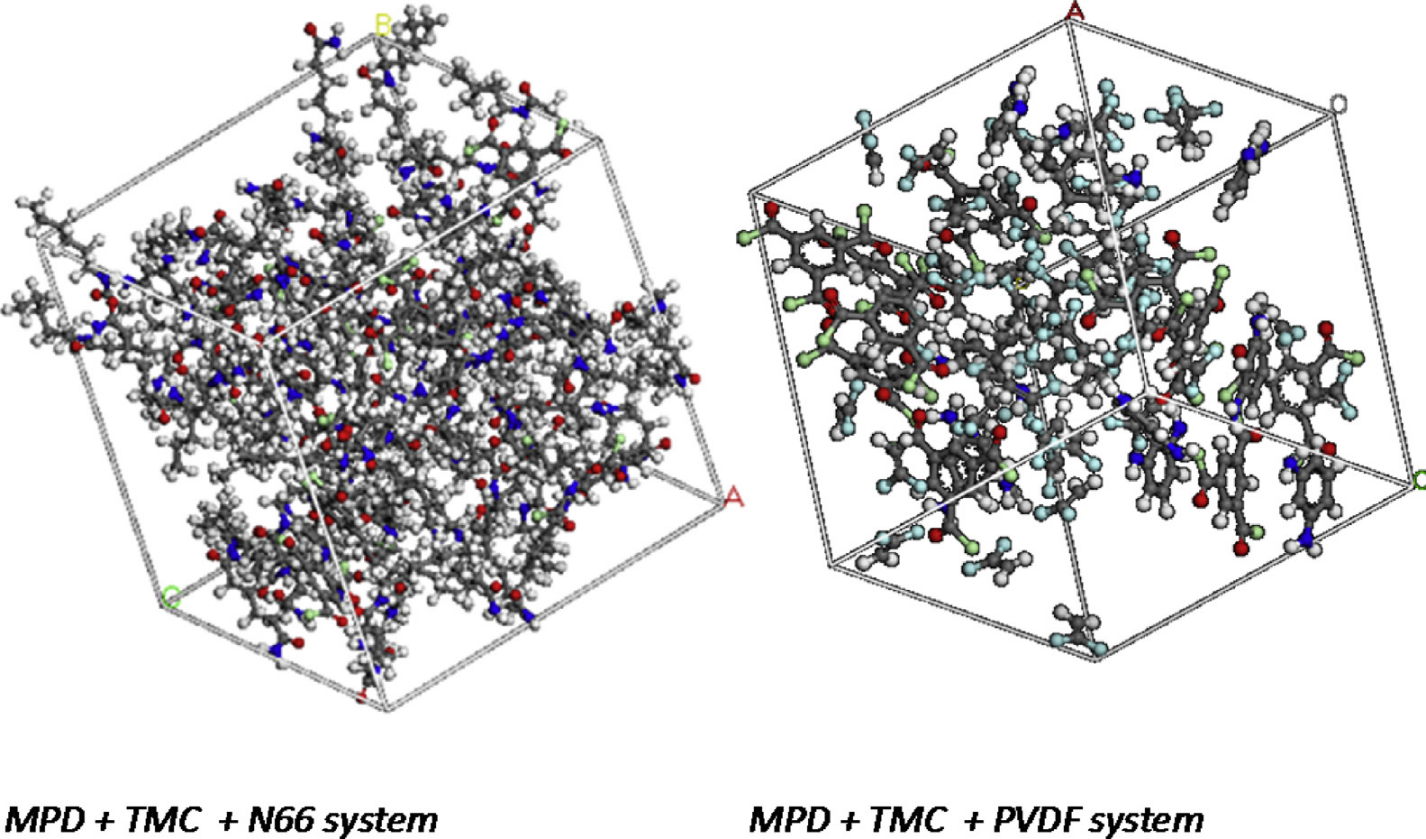
3D boxes representing the simulations.

All simulations were performed using Material Studio (version 7.0) software from Accelrys, Inc. Models were firstly undergoing the geometry optimization and then the energy started to minimized. The molecules of the models chosen represent in Fig. 4. This process repeated until convergence of charge and energy was completed. Minimizations stages were accomplished operating the Smart Minimization mode that switches from steepest-descent to conjugated gradient and then to the Newton–Raphson method as the energy derivatives decrease in order to speed the computation [14], [15]. For each polymer, the repeating unit was first built and its geometry optimized by energy minimization using the COMPASS (condensed-phase optimized molecular potential for atomistic simulation studies) force field [16], [17]. Then, the amorphous cell module was employed to fold one aromatic polyamide chain with repeating units into a periodic unit cell at ambient temperature to generate 10 configurations shown in Fig. 5. Then, the configurations were employed an energy minimization process using the followed by NVE (number molecules, volumes, and total energy) and NPT (number molecules, pressure, and temperature) ensemble at 1 atm according to the protocol described by in order to obtain an optimized polymer cell [18]. Each NVE and NPT ensembles have performed a total of 1000ps simulation time-step. The final simulation trajectory data was analysed by RDF [19].
