# Supplementary material for: Data for molecular recognition between polyamide thin film composite on the polymeric subtract by molecular dynamic
Source: Data Brief. 2019 May 3;24:103910. doi: 10.1016/j.dib.2019.103910 (PMC6535686; doi:10.1016/j.dib.2019.103910)
Supplement: Multimedia component 1 [file mmc1.doc]

Conflict of Interest and Authorship Conformation Form

Please check the following as appropriate:

- All authors have participated in (a) conception and design, or analysis and interpretation of the data; (b) drafting the article or revising it critically for important intellectual content; and (c) approval of the final version.
- This manuscript has not been submitted to, nor is under review at, another journal or other publishing venue.
- The authors have no affiliation with any organization with a direct or indirect financial interest in the subject matter discussed in the manuscript
- The following authors have affiliations with organizations with direct or indirect financial interest in the subject matter discussed in the manuscript:

| Corresponding author’s name | Affiliation | Signature |
| --- | --- | --- |
| Sunarti Abdul Rahman | Faculty of Chemical and Natural Resources Engineering, Universiti Malaysia Pahang, 26300, Gambang, Kuantan Pahang Malaysia |  |
